# Characterization of Functional Components in Bovine Colostrum That Inhibit Norovirus Capsid Protruding Domains Interacting with HBGA Ligands

**DOI:** 10.3390/pathogens10070857

**Published:** 2021-07-07

**Authors:** Zhaolei Xue, Qi Han, Pengwei Huang, Xi Jiang, Ming Tan, Yaofeng Zhao, Ning Li, Ran Zhang

**Affiliations:** 1 State Key Laboratory of Agrobiotechnology, College of Biological Sciences, China Agricultural University, Beijing 100193, China; zhaolei874@163.com (Z.X.); sz20153020062@cau.edu.cn (Q.H.); 06003h@cau.edu.cn (Y.Z.); ninglicau0709@gmail.com (N.L.); 2 Division of Infectious Diseases, Cincinnati Children’s Hospital Medical Center, Cincinnati, OH 45229, USA; Pengwei.huang@CCHMC.org (P.H.); Jason.jiang@cchmc.org (X.J.); Ming.Tan@cchmc.org (M.T.); 3 Department of Pediatrics, University of Cincinnati College of Medicine, Cincinnati, OH 45229, USA

**Keywords:** human norovirus, virus–host interaction, norovirus receptor, bovine colostrum (bCM), antiviral

## Abstract

Human noroviruses (huNoVs) cause epidemic acute gastroenteritis with significant mortality and morbidity worldwide. However, there are no commercial vaccines or antivirals against these important pathogens so far. In this study, we found that bovine colostrum (bCM) inhibited huNoV VLPs and their capsid-protruding (P) domains binding to histo-blood group antigens (HBGAs) that are huNoV receptor or attachment factors for infection, suggesting that bCM may function as a natural antiviral against huNoVs. We then characterized the bCM for the functional inhibition components by sequentially separating bCM into multiple fractions through various chromatography approaches, followed by determining their inhibitory abilities against huNoV receptor-binding P protein interacting with HBGAs. The protein components of bCM functional fractions were examined by two-dimensional polyacrylamide gel electrophoresis (2D-PAGE). Our data suggested that some milk proteins, likely in the form of glycoproteins, contribute to the observed blocking effects of bCM. Our findings lay an important foundation to further develop bCM into a potential natural antiviral against huNoVs.

## 1. Introduction

Human noroviruses (huNoVs), members of the *Norovirus* genus in the calicivirus family, are the most important viral pathogens of acute epidemic gastroenteritis. HuNoVs are highly contagious, infecting millions of people worldwide annually, claiming 218,000 lives with significant morbidity and enormous economic loss [1]. In the United States alone, huNoVs cause about 20 million infections with 570–800 deaths per annum [2,3]. HuNoVs transmit via the fecal–oral route and through person-to-person contacts with an estimated minimum 50% infectious dose of 18 virus particles [4]. As a result, huNoVs often lead to large outbreaks of acute gastroenteritis in a wide variety of close or semi-close settings, including cruise ships, large battle ships, military bases, hospitals, restaurants, and schools [5,6,7]. While huNoV-caused diseases are generally self-limited, severe diarrhea, prolonged symptom duration, and infection complications can be lethal to the elderly population, children, immunity compromised patients, and others in poor health status [3,8,9]. Currently, there are no commercial vaccines or antivirals against these important pathogens; however, the increased numbers of huNoV outbreaks in various facilities highlight an urgent need of an effective solution to control and prevent huNoV infections and illnesses.

HuNoVs recognize histo-blood group antigens (HBGAs) as host attachment factors or receptors that play an important role in huNoV host susceptibility and host ranges (reviewed in [10,11]). HBGAs are fucose-containing glycans that distribute on red blood cells as determinants of our blood types, including various ABO, Lewis, and secretor/nonsecretor types [12]. HBGAs also distribute abundantly on the mucosal surface of our intestinal tracts, where they serve as attachment factors or receptors for huNoV infection (reviewed in [10,11]). In addition, HBGAs are present in biological fluids, such as saliva and milk, that serve as reagents for in vitro assays of huNoV–HBGA interactions. The biosyntheses of HBGAs are catalyzed by specific glycosyltransferases to sequentially add individual saccharides to the HBGA precursors, producing various HBGA end products.

As non-enveloped RNA viruses, huNoVs are capsulated by an icosahedral protein capsid that is composed of 180 capsid protein or VP1 of huNoVs. Each capsid protein can be divided into two major domains: the N-terminal shell (S) domain forming the interior shell, and the C-terminal protruding (P) domain that builds the protrusion dimers of the capsid [13]. The P domain is responsible for huNoV–host interactions, binding the HBGA ligands or receptors to initiate huNoV infection [14]. In vitro expression of the P domains form P domain dimers that retain the authentic structure and HBGA-binding function as the huNoVs [15,16,17]. Production of modified P domains also self-assembled into different P domain particles or complexes, including the P_24_ particles [18,19], the P_12_ particles [20], and various polymers [21,22] with HBGA-binding functions. Thus, P domains are excellent models to study huNoV–host interactions. They are particularly useful because huNoVs cannot be efficiently cultivated in a conventional cell culture system and a small animal model for the huNoV challenge remains lacking.

Milk, particularly colostrum that is produced in the first week after parturition, provides infants both nutrition and immune protection against various pathogens [23]. Bovine colostrum milk (bCM) is rich in immunoglobulins [24,25], lactoferrin [26,27], free oligosaccharides, oligosaccharide-containing glycoproteins [28,29], and lactoperoxidase [30]. In fact, bCM has been used to treat acute diarrhea [31,32] and other gastrointestinal infections [33,34,35]. BCM was also shown to reduce huNoV VLP binding to human intestinal Caco-2 cells [36]. Further studies showed that lactoferrin, a multifunctional, globular glycoprotein in bCM, inhibited infection and replication of two huNoV surrogates, murine NoVs (MNV), and feline calicivirus (FCV) in cell culture [37,38]. Our previous study also showed that certain high molecular weight components from secretor human milk, likely to be glycoproteins, blocked huNoV VLPs attached to their HBGA ligands [39,40]. Another study showed that the IgG component of bCM reacted with huNoV VLP, suggesting that the cross-reactive antibodies in the bCM may be a functional component to block huNoV VLP to intestinal cells [36].

In this study, we observed that bCM strongly blocked the interactions of huNoV VLPs and the huNoV receptor-binding P domain with their HBGA ligands. Further characterization of bCM components via various chromatography methods, followed by testing their blocking effects against huNoV P proteins interacting with HBGAs, showed that the functional components occurred in multiple fractions with distinct milk proteins. Thus, multiple components of bCM, probably glycosylated proteins, contribute to the observed blocking effects. Our data lay a solid basis for the future development of bCM into a potential natural antiviral against huNoVs.

## 2. Results

### 2.1. Blockade of BCM against HuNoV VLPs Binding to HBGAs

**Figure 1:**
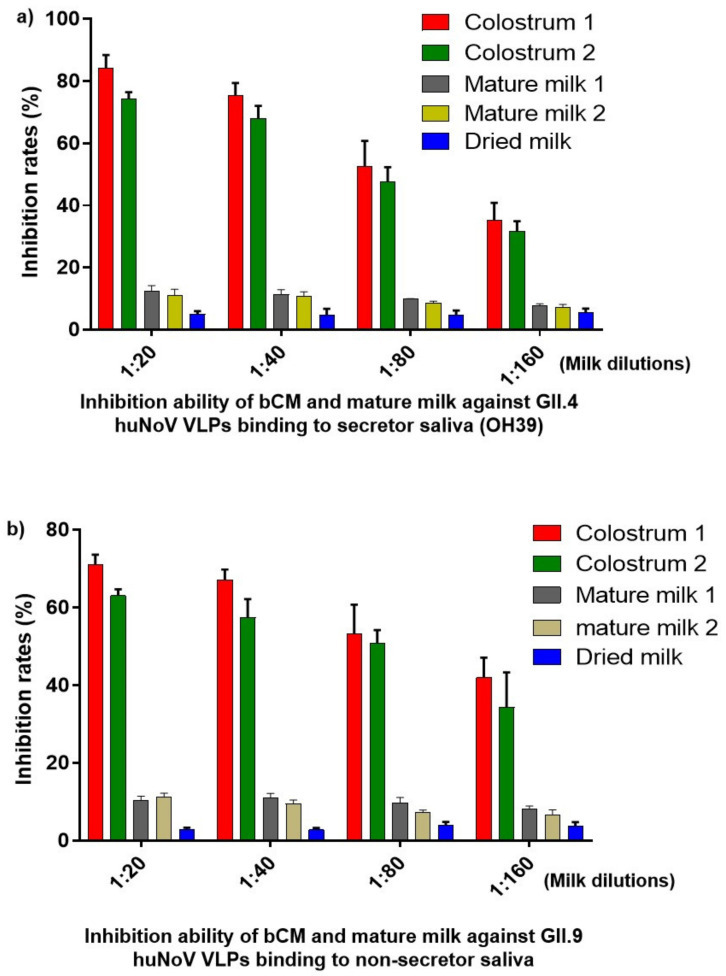
Bovine colostrum (bCM) strongly blocked attachment of human norovirus (huNoV) VLPs to HBGA receptors. (**a**) Two bCM samples inhibited VLPs of huNoV GII.4 VA387 strain binding to a saliva sample with secretor histo-blood group antigen (HBGA) types. (**b**) The same two bCM samples also blocked VLPs of huNoV GII.9 VA207 strain binding to a saliva sample with nonsecretor HBGA types. x-axis indicates dilutions of bCM, mature milk, and dried milk; mature milk and dried milk samples are controls for comparisons. y-axis indicates their inhibition rates.

Our study started with the observations that bCMs strongly blocked huNoV VLPs binding to HBGA ligands in a dose-dependent manner (Figure 1), showing over 50% inhibition rates at 1:80 bCM dilution. These inhibitory effects occurred to both huNoV GII.4 VA387 strain that binds to secretor HBGAs (Figure 1a) and GII.9 VA207 strain that binds nonsecretor HBGAs (Figure 1b). Mature bovine milk samples also exhibited low inhibitory effects that were significantly weaker than those by the bCM samples (all *Ps* < 0.01). These data suggested that bCMs may be an effective antiviral candidate against huNoVs, and it is of significance to further characterize functional components of bCM.

### 2.2. Validation of HuNoV P Proteins as a Tool to Study BCM–HBGA Interaction

**Figure 2:**
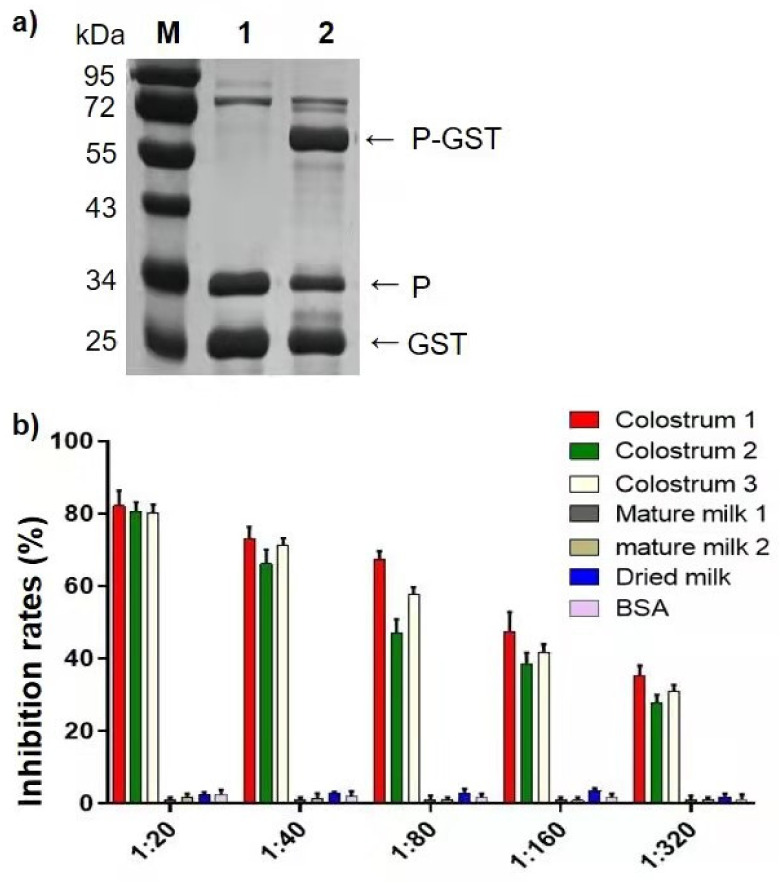
Validation of huNoV P–GST fusion proteins as a tool to study huNoV-–HGBA interactions. (**a**) SDS-PAGE showing *E. coli*-expressed GII.4 P–GST fusion protein. Lane 1, purified P–GST fusion protein after cleavage by thrombin, showing separate P protein and GST; lane 2, affinity column-purified P–GST fusion protein. The three motioned proteins are indicated by arrows. Lane M, protein standards with indicated protein sizes. (**b**) Three bovine colostrum milk (bCM) samples showed similar blockage against GII.4 huNoV P proteins attaching to secretor HBGAs (secretor saliva sample), such as those coffered using huNoV VLPs (Figure 1a). y-axis indicates the inhibition rates. x-axis indicates the dilutions of bCM, mature milk, and dried milk; mature milk and dried milk are controls for comparisons.

Previous studies have demonstrated that recombinant huNoV P proteins that retain authentic HBGA binding function are a good tool to study huNoV–HBGA interaction [17]. For such a tool, the P–GST fusion proteins were produced (Figure 2a) to perform blocking assays. The results (Figure 2b) indicated that blocking assays using the P–GST fusion protein to replace huNoV VLPs showed very similar outcomes, validating the recombinant P–GST fusion protein as a useful model to identify the effective components of bCM that block the huNoV VLP–HBGA interaction.

### 2.3. Separations of BCM Components

**Figure 3:**
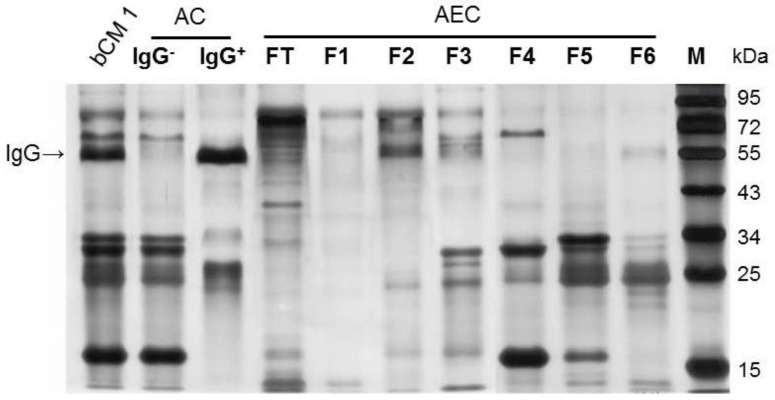
Analysis of bCM components in full bCM and its various fractions by SDS-PAGE followed by silver staining. bCM was first treated by protein A-affinity chromatography (AC) and divided into non-IgG (IgG^−^) and IgG (IgG^+^) fractions. The non-IgG (IgG^−^) fraction was then treated by an anion exchange chromatography (AEC) and divided into six fractions (lanes FT to F6). Lane bCM 1, bCM before separation; lane IgG^−^, non-IgG fraction of bCM; lane IgG^+^, IgG fraction of bCM; lane FT, AEC flow through, lanes F1 to F6, fractions 1 to 6 of AEC of bCM non-IgG portion, lane M, protein standards with indicating molecular weights in kDa. The location of IgG is indicated by an arrow. Related AC and AEC elution curves with fraction labeling are shown in Figure 4.

A bCM (colostrum 1) was separated into different fractions containing distinct components through three methods: (1) affinity chromatography using a protein A column to separate bCM into IgG and non-IgG fractions; (2) non-IgG bCM fraction were further separated into seven fractions via anion exchange chromatography (AEC) based on their electronic configurations of the bCM components; and (3) the effective AEC fractions were further separated by gel-filtration chromatography into three fractions using a size exclusion column (see Materials and Methods). The resulted fractions were analyzed by SDS-PAGE (Figure 3) or 2D-PAGE (Figure 5), followed by silver staining.

### 2.4. The BCM IgG Fraction Lacking-Blocking Effects

**Figure 4:**
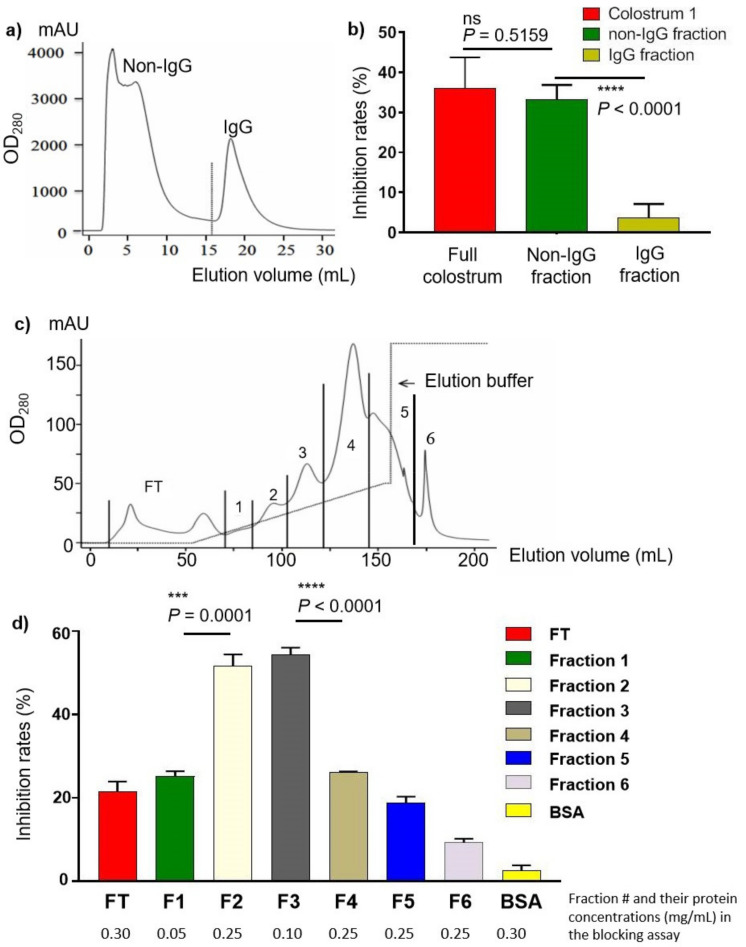
Blocking effects of various bCM fractions against huNoV P protein–HBGA interactions. (**a**,**b**) Blocking effects of IgG and non-IgG fractions of bCM against huNoV P protein–HBGA interactions. (**a**) Elution curve of an affinity chromatography using a protein-A column to divide bCM into IgG and non-IgG fractions. x-axis indicates the elusion volume, while y-axis indicates the UV_280_ absorbance values of the eluted components. (**b**) Inhibitory rates against huNoV P protein–HBGA interaction (y-axis) by the IgG (gold column) and non-IgG (red column) fractions, compared with the untreated bCM (red column) (x-axis). Around 0.25 µg/mL proteins from each sample were used in ELISA. (**c,d**) Blocking effects of various fractions of the non-IgG bCM against huNoV P protein-HBGA interactions. (**c**) Elution curve of an anion exchange chromatography (AEC) of the non-IgG bCM. Elution was divided into seven fractions: flow-through (FT), fractions 1 to 6. x-axis indicates the elusion volume, while y-axis indicates the UV_280_ absorbance values of the eluted components. (**d**) Inhibitory rates against huNoV P protein–HBGA interaction (y-axis) by various AEC fractions (x-axis). BSA was used as negative control. Protein concentrations that were used in the blocking assays were shown below each fraction. The volume of each fraction used in the blocking assays was the same. Statistical significances between major data groups are shown. ns indicate no significant differences; ***, *p* < 0.001; ****, *p* < 0.0001.

BCM is rich in IgG (Figure 3, lane IgG^+^) and a previous study suggested that bCM IgG might be a blocking factor against huNoV VLPs attaching to intestinal Caco 2 cells [36]. Thus, we first examined whether bCM IgG may play a role in the observed blocking effects. A protein-A column was utilized to separated IgG from other bCM components (Figure 4a), resulting in IgG (IgG^+^) and the non-IgG (IgG^−^) portions. SDS-PAGE analysis confirmed the good separation of IgG from the non-IgG portion (Figure 3). Blocking assays, however, showed that the IgG^+^ fraction exhibited only a marginal inhibitory effect (~3%) against P protein–HBGA interaction, which is significantly lower than that (~35%) conferred by the non-IgG fraction (Figure 4b, *p* < 0.0001). In fact, the non-IgG fraction showed a similar blocking effect as that by the bCM before IgG removal (*p* = 0.5159). Thus, we concluded that bCM IgG is not a functional component inhibiting huNoV VLP/P protein–HBGA interactions.

### 2.5. Blocking Roles of Various AEC Fractions

We then further separated the bCM non-IgG portion by AEC into seven fractions, including the flow-through (FT) and six fractions representing the six peaks of the AEC elution (Figure 4c). The fractions were determined for their protein concentrations, while their protein components were analyzed by SDS-PAGE with silver staining (Figure 3, AEC). Their blocking effects were assessed by blocking assays using the same volume amount (Figure 4d). The results showed that all fractions revealed certain blocking effects (>9%), but fractions 2 and 3 (F2 and F3) exhibited the highest blocking rates over 51%, significantly higher than those of the other fractions (*Ps* ≤ 0.0001). Due to the very low protein concentration of fraction 1 (F1), its blocking rate of 23% was considered high. Thus fractions 1, 2, and 3 (F1, F2, and F3) were selected for further study.

### 2.6. 2D-PAGE Analyses of AEC F1 to F3

**Figure 5:**
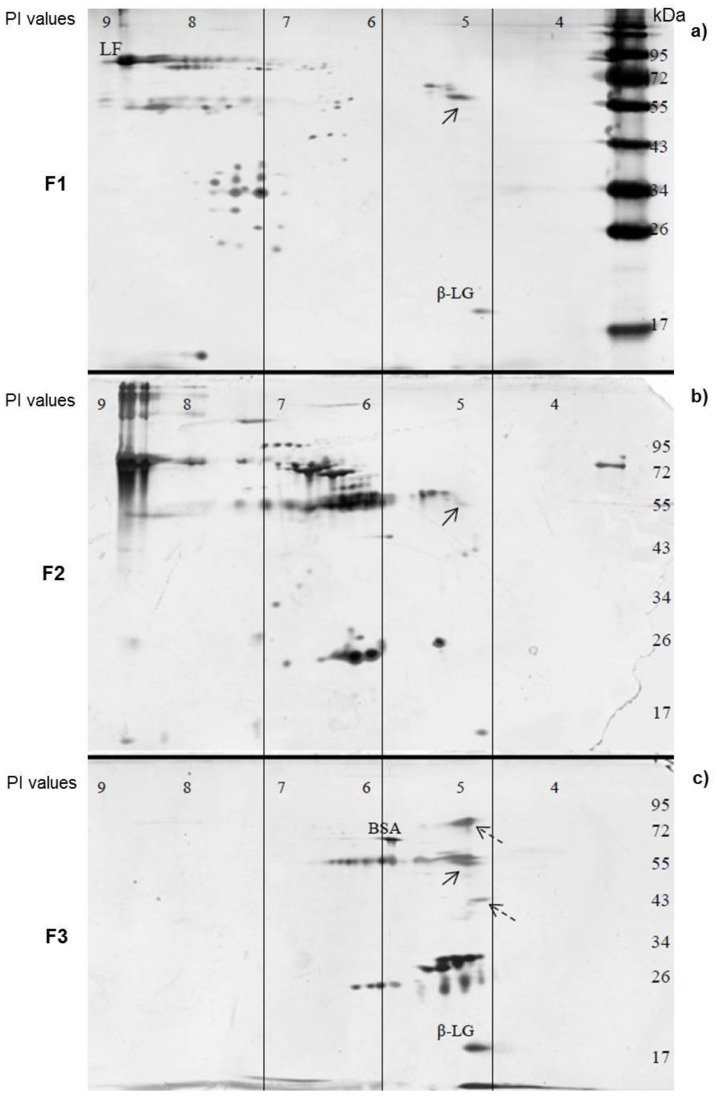
Two-dimensional (2D)-PAGE analysis of AEC fractions 1 (F1) (**a**), 2 (F2) (**b**) and 3 (F3) (**c**). Numbers on the top indicate the isoelectric point (PI) values. Three vertical lines help to locate the corresponding PI regions of the three gels. The numbers on the right indicate the molecular weights. Three typical bovine milk proteins, lactoferrin (LF, MW 78 kDa, PI 8.7), bovine serum albumin (BSA, 69 kDa, PI 5.8), and β-lactoglobulin (β-LG, MW 20kDa, PI 4.9) are indicated. A protein that occurs in all three fractions is indicated by solid arrows, while two unique proteins that occur only in fraction 3 (F3) are shown by dashed arrows.

2D-PAGE was performed to further study the protein components in AEC F1 to F3 (Figure 5). As expected, proteins in the three fractions had distinct PI values. The PI values of most F1 proteins were between pH 8 and 9; most F2 proteins had PI values between pH 6 and 7, while the majority of the F3 proteins had PI values at pH 5. Based on the known 2D-PAGE data of milk proteins [41], three typical bovine milk proteins can be recognized, including lactoferrin (LF, MW 78 kDa, PI 8.7), bovine serum albumin (BSA, 69 kDa, PI 5.8), and β-lactoglobulin (β-LG, MW 20kDa, PI 4.9). While the majority of the protein components appeared different among the three fractions, they seemed to share a common protein at about 55 kDa with a PI value of 4.9 (Figure 5, solid arrows). On the other hand, we found two unique proteins at 80 kDa with a PI of ~4.9 and 40 kDa with a PI of ~4.8 in F3 that showed the strongest blocking effects.

### 2.7. Gel-Filtration Analyses of AEC F1 to F3

**Figure 6:**
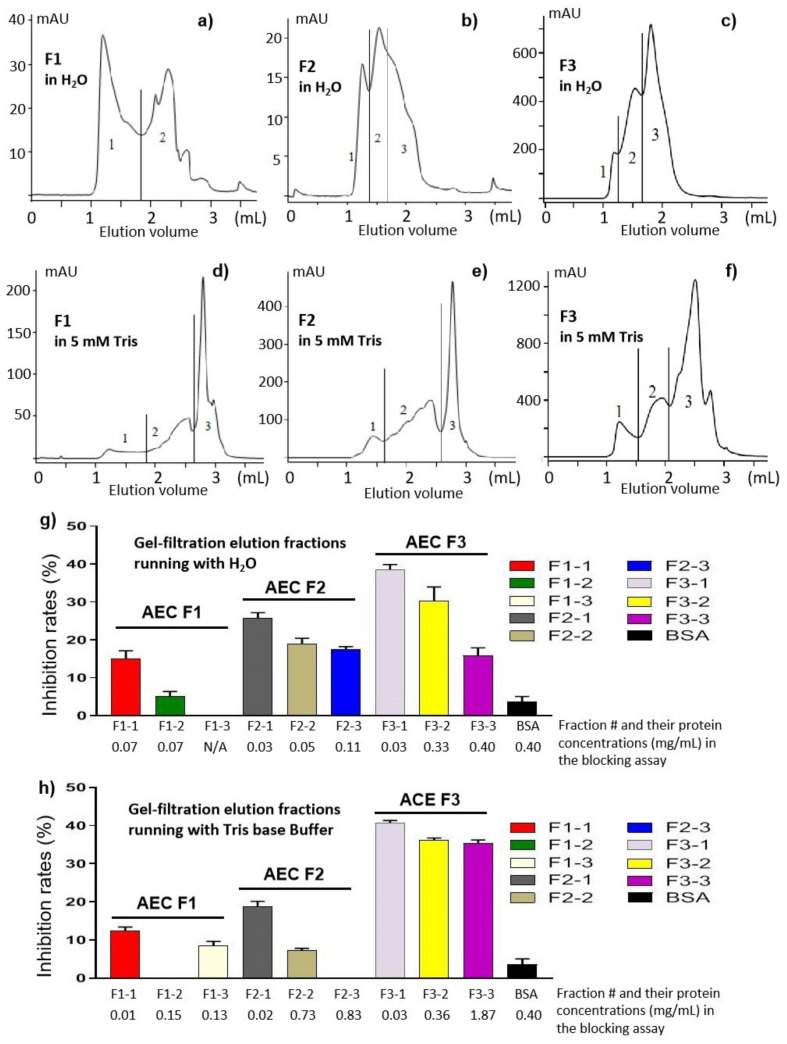
Gel-filtration analyses of the AEC F1, F2, and F3 fractions and the HBGA-binding blocking effects of the resulting fractions. (**a**–**f**) Elution curves of gel filtrations were performed using a size exclusion column (SEC) running with water (**a**–**c**) and 5 mM Tris-base (**d**–**f**), respectively. Each elution was divided into three fractions covered typical elution peaks. x-axis indicates the elution volume, while y-axis indicates the UV_280_ absorbance. (**g**,**h**) Blocking effects of the elution fractions against huNoV P protein binding to the HBGAs. The protein concentrations of each fraction were determined and indicated to each fraction. BSA was used as a negative control.

The gel-filtration chromatograph approach was used to further separate the components of the AEC F1 to F3 based on their molecular weights using a size exclusion column (SEC). As a preparation for the downstream study by 2D-PAGE, gel filtrations were performed using water (Figure 6a–c) and low salt concentration buffer of 5 mM Tris-base (Figure 6d–f), respectively. We noted that the UV_280_ readings (mAU) in the gel filtrations running with water were lower than those of gel filtrations running with the 5 mM Tris base buffer, suggesting that some proteins may precipitate in water. This may also explain the differences regarding the elusion peaks of the gel filtrations using the two running solutions (Figure 6a–c compared with Figure 6d–f). Each elution was divided into three (two for F1 in H_2_O) fractions that covered typical elution peaks (Figure 6a–f).

The fractions were determined for their protein concentrations (data not shown) and examined for their blocking effects against huNoV P protein binding to the HBGA ligands (Figure 6g,h), revealing the following results. First, the highest blocking activities were seen in the earliest eluted fractions (F1-1, F2-1, and F3-1), even though these fractions contained the least protein amount in general, as seen in the elusion curves of the gel filtrations (Figure 6a–g). Thus, the functional factors should be associated with large molecules or polyvalent complexes in high molecular weights. Second, blocking effects were not observed in a single fraction; instead, they appeared to occur in multiple fractions, suggesting that the functional factors may be associated with multiple proteins. Thus, all gel-filtration fractions will be further analyzed by 2D-PAGE, focusing on the AEC F3 that exhibited the strongest blocking effects.

### 2.8. 2D-PAGE Analysis of the Gel Filtration Fractions

**Figure 7:**
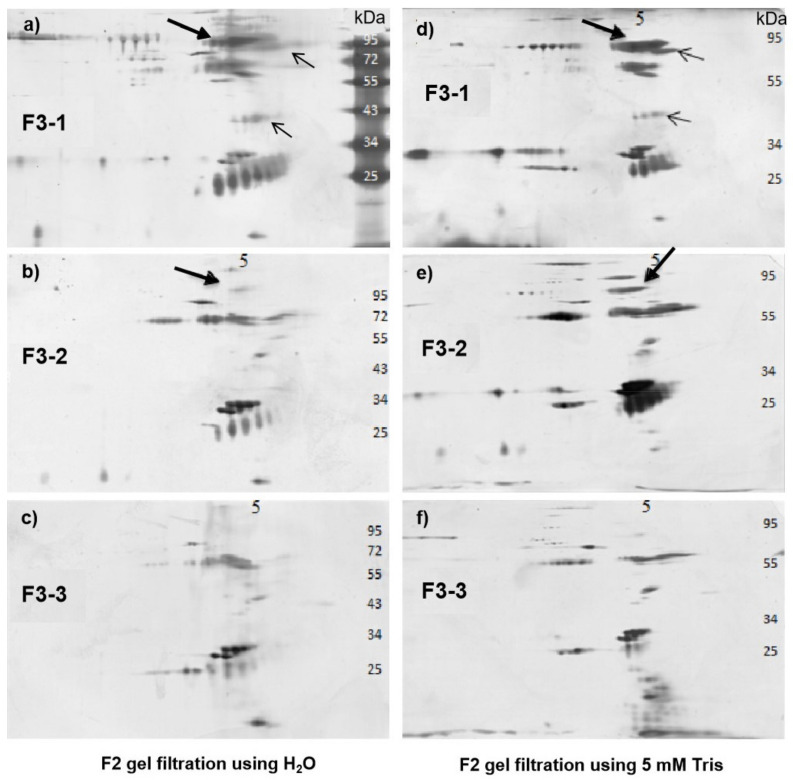
Two-dimensional (2D)-PAGE analysis of the six gel-filtration fractions of AEC F3. (**a**–**c**) 2D-PAGE analysis of the three fractions of gel-filtration running with water (left panel). (**d**–**f**) 2D-PAGE analysis of the three fractions of gel-filtration running with 5 mM Tris-base buffer (right panel). Numbers on the top indicate the isoelectric point (PI) values. The numbers on the right indicate the molecular weights. The proteins (84 kDa, PI: ~5.5) that occurred in AEC F3-1 and F3-2 fractions but not in F3-3 fractions are indicated by bold arrows. Two other proteins that occur only in F3-1 fractions are indicated by non-bold (fine) arrows.

The six gel-filtration fractions of AEC F3 in H_2_O and Tris buffer, respectively, were analyzed by 2D-PAGE (Figure 7), while the gel-filtration fractions from AEC F1 and F2 were also analyzed (Supplementary Materials Figures S1 and S2) for comparisons. Again, multiple proteins were seen in each fraction, making it hard to claim which protein(s) played major roles in the observed blocking effects of bCM at this time. However, we did note that a protein at a large amount at 84 kDa with PI of 5.5 occurred in F3-1 and F3-2 fractions, but not in F3-3 fractions (Figure 7a,b,d,e, solid arrows). This protein at much less amount was also present in F1-1 and F2-1 fractions (Supplementary Materials Figures S1 and S2), but not at F1-2, F1-3, F2-2, and F2-3 fractions. Two other unique proteins were also noted to be in the F3-1 fractions (Figure 7a,d, fine arrows), but not in other F3 fractions. Finally, a unique protein at 55 kDa with PI of 5 was seen in most of the F1 and F2 gel-filtration fractions (Supplementary Materials Figures S1 and S2, dashed arrows). In summary, our data suggest that the effective bCM components may not be a single protein type, but a group of protein-associated components in large but different molecular weights.

## 3. Discussion

In addition to its nutrition roles, bCM is known for its protection for infants against various enteric pathogens [23]. In this study, we found that bCM blocks two types (GII.4 and GII.9) of huNoV VLPs binding to their viral receptors, suggesting that bCM may function as a natural antiviral against huNoVs, an important step to understand the mechanism behind the observed antiviral effects of bCM for future development into a potent antiviral against huNoVs. We set off to study the observed blocking effects, aiming to identify the functional bCM components. To this end, bCM was serially separated into multiple fractions with different components via various chromatography approaches, followed by the determination of their blocking effects against huNoV P proteins binding to their HBGA ligands. We found that the blocking components occurred in multiple fractions, suggesting that there is not a single but multiple bCM molecules that contribute to the observed bCM blocking effects.

HuNoVs recognize HBGA glycans on the mucosal surface of the intestine as receptors or attachment factors to initiate viral infections [10]. The HBGA binding sites (HBSs) have been shown to be located on the outermost surface of the capsid P domains of huNoV virions. Based on the known information of huNoV–HBGA interaction [11,14], bCM components could inhibit such an interaction in two possible ways. First, the functional bCM components can be a protein that interacts with the P domain protein and thus cover, destroy, and/or indirectly affect the function of huNoV HBSs. If this is the case, one should expect that only a single or very few unique bCM proteins can interact with huNoV P proteins. However, our results showed that multiple protein fractions exhibited such blocking effects, strongly suggesting that it is not a single protein contributing to the observed blocking effects.

Thus, the second possibility becomes more likely. In this case, the functional bCM factors may be a group of HBGA- or glycan-containing proteins, namely, glycoproteins, that interact with the HBSs of huNoV P proteins through their HBGA/glycan moieties. Milk is known for its enrichment of free oligosaccharides and glycoproteins [28,29,42]. However, although free oligosaccharides may be able to bind the HBSs, they may not play a key role in the observed blocking effects due to their small molecular sizes and low valences, resulting in low avidity. In addition, free oligosaccharides should be concentrated in the low molecular fractions of a gel-filtration chromatography, but we did not see high blocking activities associated with the low molecular weight fractions. By contrast, the high blocking activities were observed to be associated with the high molecular weight fractions. It is likely that the HBGA-like glycans conjugated to various proteins with high molecular sizes repeatedly, leading to polyvalent and exhibiting high binding avidity to the HBSs of the P domain proteins. This scenario explains why the effective components occur in multiple fractions and the blocking activity was associated with fractions of high molecular weights, as observed in this study. In fact, the large molecular fractions of milk from a gel-filtration chromatography have been shown to be recognized by various HBGA-specific monoclonal antibodies and bind various huNoV VLPs [40]. Thus, we concluded that it is highly possible that the large glycoproteins in the high molecular fractions of gel filtrations contain polyvalent HBGA-like glycans and thus bound and blocked the function of huNoV HBSs.

Another previous study [36] observed that the IgG of bCM reacted with huNoV VLPs and thus suggested IgG may be the functional component to inhibit the observed huNoV VLP–Caco 2 cell interaction. However, our study did not support this observation, because the bCM fraction in our study (Figure 3, lane IgG^+^) did not block the huNoV P protein–HBGA interaction (Figure 4a,b). One possible explanation is that the bCM IgG in the previous study may be glycosylated, while the bCM IgG in our study did not. This issue needs to be clarified by further study.

In addition, other studies demonstrated that bCM lactoferrin, a multifunctional glycoprotein, reduced replication in cell culture of MNV and FCV [37,38] that are two huNoV surrogates. It should be noted that MNV and FCV are known to recognize sialic acids, not HBGAs as receptors [43,44]. In fact, there are many possibilities as to how lactoferrin can interfere with viral infection in general. A recent NMR study showed that MNV P dimers bind to neither HBGAs nor sialoglycans [45]. This explains why there is no solved structure for complexes of MNV P dimers with sialylated glycans, whereas there are plenty of crystal structure data for huNoV P dimers complexed with HBGAs. Therefore, previously observed sialoglycan–MNV interactions may be attributed to "indirect" causes. Even the bCM lactoferrin was shown to block replication of MNV and FCV; we did not observe an association between the amount of lactoferrin and the blocking effects, because the F1 fraction of gel filtration contained the most lactoferrin amount but exhibited only low blocking effects, while the F3 fraction lacked lactoferrin, but showed the strongest blocking effects (Figure 4d; Figure 5 compared a and c). Thus, while further study is necessary to clarify the mechanism behind the observed blocking effects, we hypothesized that glycosylated proteins with HBGA-like glycans in bCM might be the major effective factors blocking the observed huNoV VLP/P protein–HBGA interaction.

## 4. Materials and Methods

### 4.1. Mature Bovine Milk and bCM Preparation

Both mature bovine milk and bCM samples were collected from Holstein cows, bCM samples were collected on day 3 after parturition or prolactin treatment, while mature milk samples were collected at least 3 weeks after parturition or prolactin treatment. The milk samples were stored at −80 °C. For further treatment, the milk samples were thawed at 4 ℃ overnight and centrifuged at 2000× *g* for 20 min at 4 °C using Optima™ L-100XP centrifuge (Beckman, Brea, CA, USA). Fluid fraction in the middle was recovered, while the fat layer on the top and the precipitates were discarded. The recovered fluids were centrifuged again at 40,000 rpm for 1 h at 4 °C to separate whey and casein fractions that were then stored at −80 °C until use. Protein concentrations were determined by bicinchoninic acid (BCA) method.

### 4.2. Production of HuNoV Virus-Like Particles (VLPs) and P Domain Proteins

Various recombinant huNoV VLPs were from our lab stocks that were made in our previous study [46]. Recombinant P domain protein of a genogroup II, genotype 4 (GII.4) huNoV (strain VA387) was expressed as GST-tagged proteins (P–GST) as described previously [47], with minor modifications. The P–GST fusion proteins were purified using Pierce GST Spin Purification Kit (Thermo Fisher Scientific, Shanghai, China) and quantified by Braford method. The GST tag was removed from the P proteins after thrombin (Sigma-Aldrich, Taufkirchen, Germany) cleavage according to manufacturer’s protocol. The purified P–GST fusion proteins and P proteins were stored at −80 ℃.

### 4.3. Saliva Samples

Well-characterized saliva samples with known HBGA types were from our lab stocks collected for previous studies [46,48]. Two types of saliva samples were used. One (OH39) was secretor-type saliva that is positive for H type 1 (H1), H2, and Lewis y (Le^y^), but negative for Le^a^ and Le^x^ antigens. This saliva sample has been shown to bind GII.4 huNoV (VA398) VLPs and P proteins previously [17,46]. The other one (OH20) was a nonsecretor type saliva that is positive for Le^a^ and Le^x^, but negative for all H-related secretor type antigens that has been shown to bind GII.9 (VA207) VLPs and P proteins [16,46].

### 4.4. HuNoV VLP/P Domain-HBGA Binding Assay

This was performed based on a previously established procedure with modifications [46]. Briefly, diluted saliva samples (1:1000) in PBS were coated in the microtiter plates at 4 °C overnight. After blocking by 5% nonfat milk in PBST (PBS, pH 7.4 with 0.5% tween-20), NoV VLPs at 0.25 μg/mL or P–GST proteins at 1 μg/mL concentration were added, and the bound VLP or P proteins were detected by in house-made rabbit anti-NoV-VLP polyclonal antibody [46] (for bound VLP) or rabbit anti-GST polyclonal antibody (CWbio, Beijing, China) at a dilution of 1:5000 (for bound P–GST fusion proteins), followed by incubation with horse-radish-peroxidase-(HRP)-conjugated goat anti-rabbit secondary antibody (1:10,000). The incubation time for each step was 1 h at 37 °C. Color reactions were developed by TMB kit (CWbio), and the signals in optical density (OD) at 450 nm were measured by a spectrum reader (Spectra max 384 plus, Molecular Devices, San Jose, CA, USA). The P–GST protein, but not the GST protein, has been shown to bind HBGA previously, similar to the P protein [17].

### 4.5. Blocking Assays against HuNoV P Domain-HBGA Binding

To determine the blocking ability of bCM/mature cow milk or separate components of bCM, above binding assay procedure will be used, except that P–GST proteins were mixed with milk or milk components before the proteins were added to the wells with coated saliva samples. The blocking rates (BRs) were calculated by the following equation: BR = (OD_unblocked control_ − OD_blocked test_)/(OD_unblocked control_ − OD_negative control_) × 100%. OD_unblocked control_ was OD of the binding between saliva and VLP/P–GST protein without mixing with milk or milk components, while OD_negative control_ was binding signals of saliva to GST only.

### 4.6. Gel Filtration Chromatography

The bCM wheys were fractionated based on their molecular weights through a gel filtration using AKTA Fast performance Liquid Chromatography (FPLC) system (GE Healthcare, Watertown, MA, USA) at room temperature. Milk whey samples were thawed on ice and then filtered through 0.22 μm membrane (Millipore, MA, USA) to remove large particles or precipitants before loading on the size exclusion column (SEC, Superdex 200 5/150 GL, GE Healthcare). Eluents were fractionated according to OD_280_ peaks, and all peaks were recovered and stored at −80 °C.

### 4.7. Affinity Chromatography

A protein-A column (HiTrap Protein A HP, 5 mL, GE Healthcare) was used to separate IgG from other protein components. Milk samples were diluted to 10 mg/mL with binding buffer containing 50 mM Tris-base (pH 7.4) before loaded (1 mL). The IgG was eluted in elution buffer containing 50 mM sodium citrate (pH 4.0), and this low pH buffer was then changed into TBS containing 50 mM Tris-base and 0.15 M NaCl (pH 7.4) by dialysis at 4 °C. Proteins with molecule weights bigger than 3500 Dalton were retained.

### 4.8. Anion Exchange Chromatography (AEC)

The IgG-stripped samples collected after AC treatment was further fractionated using a DEAE weak anion exchange column (HiPrep DEAE FF 16/10, GE Healthcare). The binding buffer was 50 mM Tris-base (pH 7.4), while elution buffer contained 50 mM Tris-base and 1 M NaCl (pH 7.4). Fractionated samples were concentrated by dialysis against polyethylene glycol with molecular weight 8000 Dalton (PEG 8000), and some samples were diluted by TBS if precipitants were seen. Components in the AEC fractions may be further separated by size exclusion chromatography (SEC) using a Superdex 200 SEC column (5/150 GL, GE Healthcare) (see above).

### 4.9. Sodium Dodecyl Sulfate–Polyacrylamide Gel Electrophoresis (SDS-PAGE) and Silver Staining

Proteins of milk samples were analyzed by SDS-PAGE as described previously [42,47]. After electrophoresis, gels were stained using the Silver Stain Plus Kit (Bio-Rad, Shanghai, China) according to the instruction of the manufacturer.

### 4.10. Two-Dimensional Polyacrylamide Gel Electrophoresis (2D PAGE) and Silver Stain

All the reagents for this procedure were purchased from GE Healthcare unless otherwise indicated. Samples were dialyzed against ultrapure water to remove extra ions before loading to immobilized pH gradient (IPG) strips (7 cm, pH 4–7 or pH 3–10). Samples containing 0.5–10 μg proteins were loaded. The rehydration was processed for 12–16 h using rehydration buffer, covered by oil to prevent liquid loss. 10 mL rehydration buffer containing 4.2 g urea, 1.52 g thiourea, 0.4 g CHAPS, and 100 μg bromophenol blue; DTT and pharmalyte (pH 4–7 or pH 3–10) were added to concentrations of 0.01 g/mL and 5 μL/mL, respectively, before use.

The first-dimensional isoelectric focusing (IEF) was run using Etan IPG phor3 via following procedure: 50 V, 1 h; 100 V, 1 h; 200 V, 1.5 h; 1000 V, 2.5 h; 8000 V, 5 h; 8000 V, 5 h (hold); and 500 V, 2 h (hold). The type of unindicted steps was liner (different from “hold”). The IPG strips were used immediately or stored carefully at −20 °C until use. The second-dimension SDS-PAGE was run using a regular electrophoresis system (Bio-RAD), and protein spots were visualized by silver stain (Bytotime, Shanghai, China) according to the manufacturer’s instruction. Images of the stained gel were taken by Gel Logic 212 (Kodak, Rochester, NY, USA).

### 4.11. Statistical Analysis of Data

All data were analyzed using software SPSS 20, presented as the means ± standard error. Statistical significance among data groups was calculated by one-way analysis of variance (ANOVA) or *t*-test, in which *p* values are smaller than 0.05 (<0.05) were considered statistically significant. The data for molecular protein weights and isoelectric point (PI) values were checked using UniProt.

## Supplementary Materials

The following are available online at https://www.mdpi.com/article/10.3390/pathogens10070857/s1, Figure S1: Two-dimensional (2D)-PAGE analysis of the five gel-filtration fractions of AEC F1. Figure S2: Two-dimensional (2D)-PAGE analysis of the six gel-filtration fractions of AEC F2.
