# Supplementary material for: Characterization of Functional Components in Bovine Colostrum That Inhibit Norovirus Capsid Protruding Domains Interacting with HBGA Ligands
Source: Pathogens. 2021 Jul 7;10(7):857. doi: 10.3390/pathogens10070857 (PMC8308730; doi:10.3390/pathogens10070857)
Supplement: Supplementary file 1 [file pathogens-10-00857-s001.zip › pathogens-1276326-supplementary.pdf]

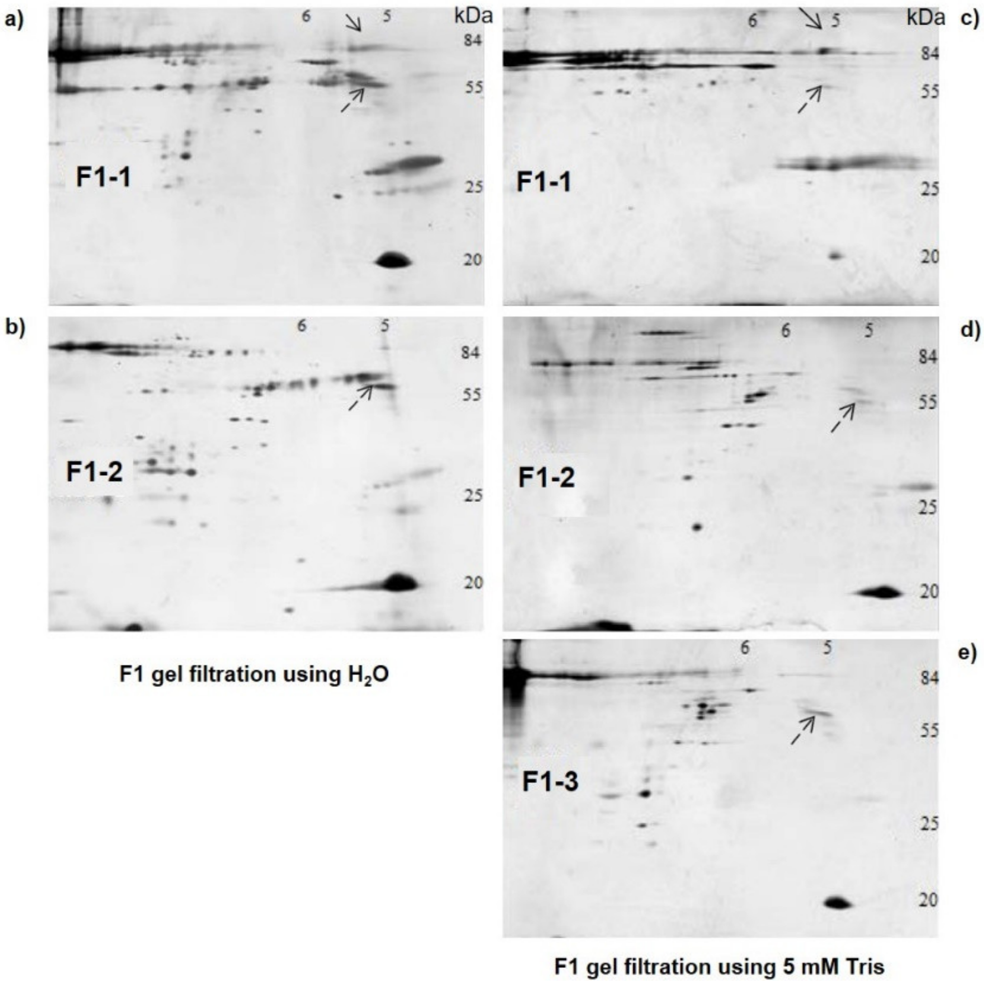

Figure S1. Two-dimensional (2D)-PAGE analysis of the five gel-filtration fractions of AEC F1. (**a**,**b**) 2D-PAGE analysis of the two fractions of gel-filtration running with water (left panel). (**c**–**e**) 2D-PAGE analysis of the three fractions of gel-filtration running with 5 mM Tris-base buffer (right panel). Numbers on the top indicate the isoelectric point (PI) values. The numbers on the right indicate the molecular weights. The proteins (84 kDa, PI: ~5.5) that occurred only in AEC F1-1 fractions, but not in F1-2 and F1-3 fractions is indicated by bold arrows. Another protein that occurs in all F1 gel-filtration fractions is indicated by dashed arrows.

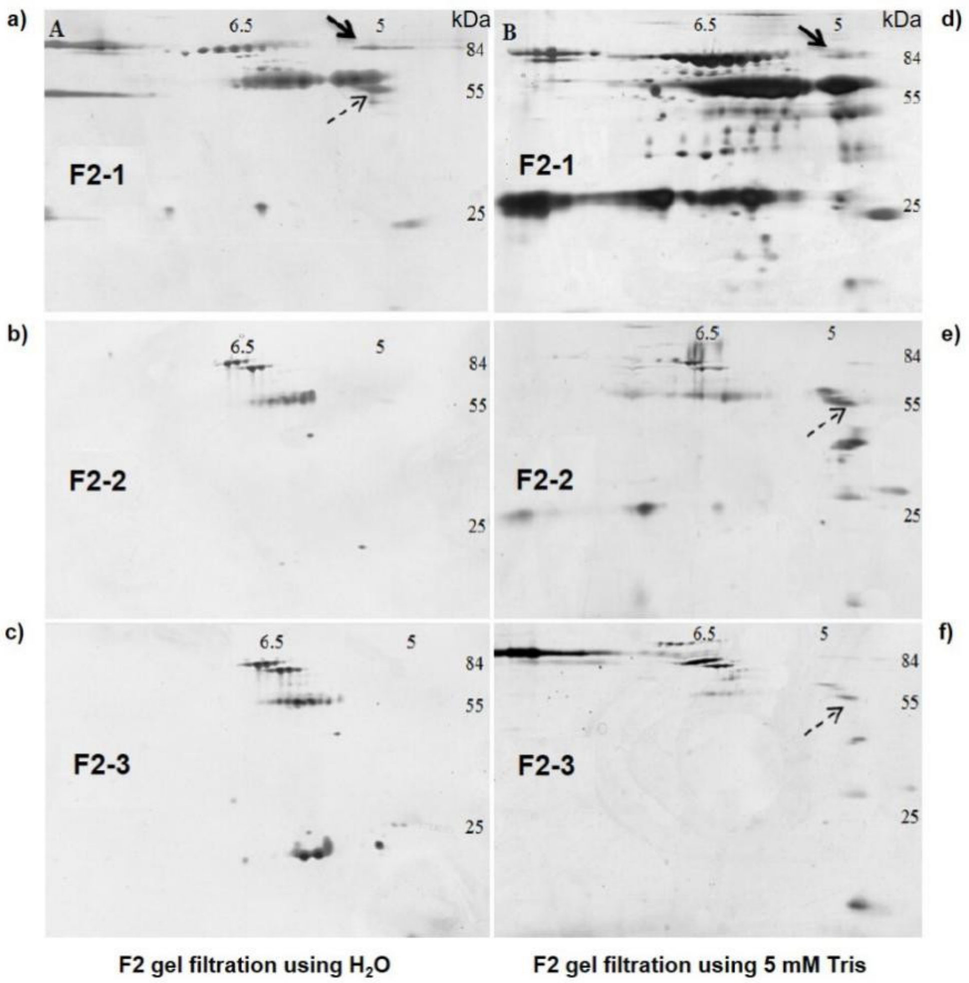

Figure S2. Two-dimensional (2D)-PAGE analysis of the six gel-filtration fractions of AEC F2. (**a**–**c**) 2D-PAGE analysis of the three fractions of gel-filtration running with water (left panel). (**d**–**f**) 2D-PAGE analysis of the three fractions of gel-filtration running with 5 mM Tris-base buffer (right panel). Numbers on the top indicate the isoelectric point (PI) values. The numbers on the right indicate the molecular weights. The proteins (84 kDa, PI: ~5.5) that occurred only in AEC F2-1 fractions is indicated by bold arrows. Another protein that occurs in most F2 gel-filtration fractions is indicated by dashed arrows.
